# Cost of image-guided percutaneous nephrostomy among cervical cancer patients at Muhimbili National Hospital in Tanzania

**DOI:** 10.1186/s12962-023-00445-9

**Published:** 2023-05-30

**Authors:** Amani Thomas Mori, Cecilia J. Nyabakari

**Affiliations:** 1 Department of Developmental Studies, Muhimbili University of Health and Allied Sciences, P.O. Box 65011, Dar es Salaam, Tanzania; 2 National Institute for Medical Research, Muhimbili Research Centre, P.O. Box 9653, Dar es salaam, Tanzania; 3 Section for Ethics and Health Economics, Department of Global Public Health and Primary Care, University of Bergen, P.O. Box 7804, Bergen, 5020 Norway

**Keywords:** Tanzania, Uterine cervical neoplasms, Costs and cost analysis, Nephrostomy, Percutaneous, Radiology, Interventional

## Abstract

**Background:**

Most cervical cancer patients in developing countries seek care in health facilities with an advanced disease, often characterized by obstructive uropathy. This study aims to estimate the cost of an image-guided percutaneous nephrostomy (PCN), which was recently introduced at Muhimbili National Hospital to manage obstructive uropathy.

**Methods:**

This was a cross-sectional study that was conducted between February and June 2021, from the provider’s perspective. The study involved forty-eight (n = 48) cervical cancer patients with obstructive uropathy. A micro-costing approach was used to identify, quantify and value both capital and recurrent cost items consumed by the patients. Cost data were collected in Tanzanian shillings and converted to USD with the relevant exchange rate. Analysis was performed in Microsoft Excel (Microsoft Excel®, Microsoft Corporation).

**Results:**

The unit cost of image-guided PCN at Muhimbili National Hospital was estimated at 380.4 USD. The main cost drivers were the single-use Nephrostomy catheters, Amplatz guide wire, and Micro-puncture set. The estimated unit cost is higher than the reimbursement price of 237.4 USD charged by the National Health Insurance Fund, and the 259.4 USD and 172.9 USD charged by the hospital for private and public patients, respectively.

**Conclusion:**

Image-guided PCN for cervical cancer patients costs three times the minimum monthly government wage. The study underscores the importance of conducting costing studies to inform pricing and reimbursement decisions in Tanzania.

## Background

Cervical cancer is caused by human papillomavirus (HPV) and is a major public health problem in the world. The primary cause of cervical cancer is persistent infection with one or more of the “high-risk” (or oncogenic) types of HPV [1]. In 2020, there were 604,127 new cases and 341,831 deaths due to cervical cancer worldwide, making it the 4th most frequently diagnosed cancer and cause of death among women [2, 3]. In 2020, Tanzania had the 4th highest incidence rate of cervical cancer in the world with 62.5 new cases and a mortality of 42.7 deaths per 100,000 women (age-standardized to the world population) [4]. The high incidence of cervical cancer prompted Tanzania to introduce the HPV vaccine in 2018 and the target is to reach 85% of the 2.7 million girls aged 14 years by 2024 [5].

Advanced cervical cancer is mostly characterized by blocked urine flow (obstructive uropathy) [6], which is among the most frequent complication found in these patients and is associated with a high mortality rate [7, 8]. Blocked urine flow may consequently cause uremia, urinary tract infections, electrolyte imbalance, and loss of renal function [9]. Unfortunately, the majority of women with cervical cancer in developing countries seek care in formal health facilities very late due to limited access to early screening and diagnosis services [7, 10]. As a result, approximately 7,000 women in Tanzania succumb to death due to cervical cancer every year because of late diagnosis and lack of effective treatment [11].

Percutaneous nephrostomy (PCN) is a recommended medical procedure to manage obstructive uropathy when retrograde drainage and surgical nephrostomy are impossible, inappropriate, or not indicated. PCN not only allows rapid recovery of renal function but also helps to clear infection thus preventing further damage [12]. Currently, PCN is performed using ultrasound and fluoroscopic imaging guidance to enhance patient safety and has an advantage over surgery because it is performed under local anesthesia [13]. Image-guided PCN is a relatively new intervention in Tanzania and is only available at Muhimbili National Hospital (MNH). The Interventional Radiology clinic that offers image-guided PCN was established at MNH in October 2018, with support from Yale University-USA. All the obstructive uropathy cases due to cervical cancer that are treated at MNH are usually referrals from Ocean Road Cancer Institute (ORCI) and other hospitals in the country.

In Tanzania, patients finance healthcare through out-of-pocket payments as private or public patients or through health insurance. Charges for public patients are subsidized by the Government while children under the age of five years, pregnant women, and the elderly above 60 years are exempted. The reimbursement unit cost for PCN by the National Health Insurance Fund (NHIF) is 237.4 USD, while MNH charges a fee of 259.4 USD for private patients and 172.9 USD for public patients. However, our literature review indicates the scarcity of economic evidence about PCN in Tanzania and Africa in general. To date, no study has been conducted to estimate the value of resources that are consumed in the provision of PCN in Tanzania. In the absence of cost information, prices set by MNH and NHIF may underestimate or overestimate the value of the actual amount of resources consumed in the procedure. Costing and cost-effectiveness studies are important to inform pricing decisions and the budget impact of scaling the intervention countrywide. Therefore, this study aims to estimate the unit cost of image-guided percutaneous nephrostomy among cervical cancer patients with obstructive uropathy at Muhimbili National Hospital in Tanzania.

## Methods

### Study area

The study was conducted at Muhimbili National Hospital (MNH) in Dar es Salaam region. Dar es Salaam is subdivided into five districts and is the largest city and business capital of Tanzania. It has a population of about 5.3 million, and a population density of more than 3100 people per km^2^, thus making it the most densely populated region in Tanzania [14]. Originally, Dar es Salaam was ethnically inhabited by the Zaramo, however, the immigration of other tribes has made the region to be culturally diverse. More than three-quarters of the residents live in informal settlements and are mostly involved in business [15]. MNH was established in 1897 and consists of different units and departments that serve referral and non-referral patients from all over Tanzania. It is also a teaching hospital and has a bed capacity of 1,500 patients. Patients with different financing modalities are treated at MNH.

### Study design

A cross-sectional costing study.

### Perspective

Provider’s perspective.

### Study population

All cervical cancer patients with obstructive uropathy referred to the MNH-IR clinic for PCN from June 2020-July 2021. Based on the records obtained from the patient register in the clinic, 48 cervical cancer patients with obstructive uropathy were treated during this period.

### Data collection methods

The study used a micro-costing/ingredients approach. The method requires meticulous identification, quantification, and valuation of all the resources consumed to provide a specific service [16, 17]. Data collection at the hospital took place from February to June 2021. We used different sources of information depending on the data type. Information about the number of patients treated came from the patient register, capital items were identified and counted, spaces were measured, and consumables were either obtained from registers or estimated for each patient by the healthcare workers. Other information about prices, salary, and salary scales was obtained from the account’s office.

**Fig. 1:**
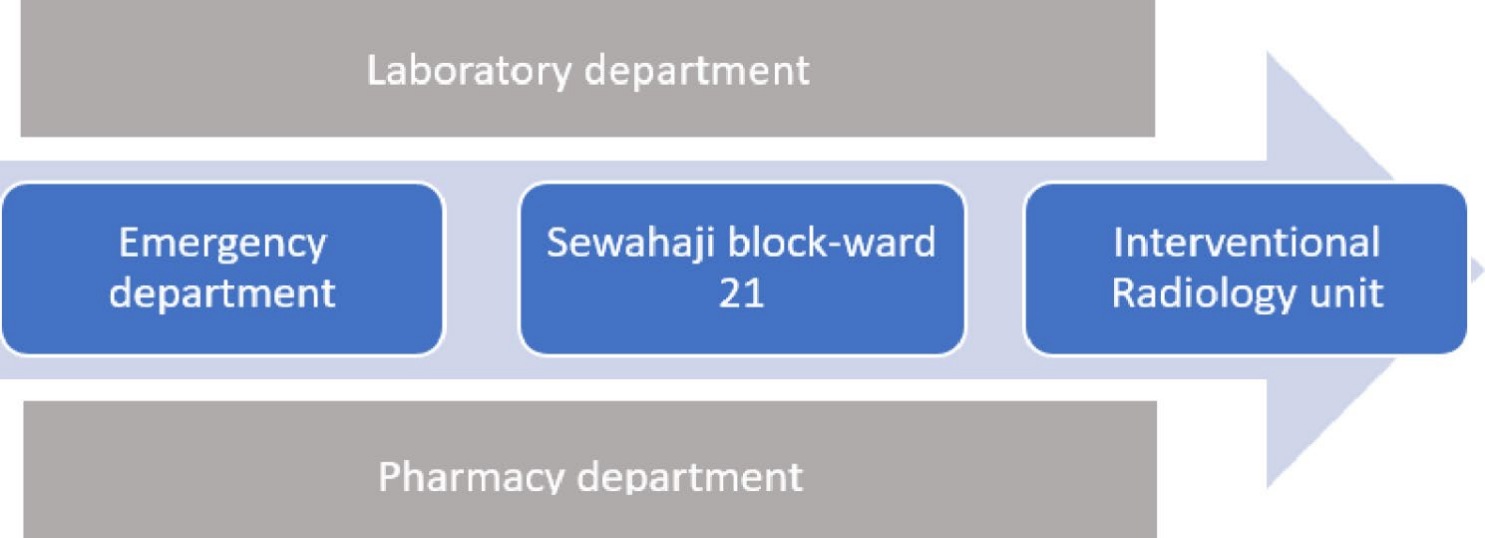
Patient pathway and cost centers

Costs data were collected from five cost centers, which were identified by tracking the patient pathways as shown in Fig. 1. These patients were first treated at the Emergency Department (EMD), before admission to ward 21-Sewahaji block. An Interventional Radiologist assessed them in ward 21 before PCN was done in the IR unit. The PCN procedure takes about two hours, and after that patients are returned to ward 21 for observation before they are discharged home the following day. Pharmacy and laboratory departments also play important roles in service provision for these patients. Usually, 4 blood samples were taken from a patient for full blood picture, serum creatinine, urea, urine, and electrolytes analyses. These samples are taken on arrival (EM department), the day after admission (Sewahaji block), and before and after performing the procedure (IR unit). Medicines, and imaging media all were supplied by the pharmacy department. The reports and registers containing utilization data and cost information about the consumable and non-consumable items in these departments were identified with the assistance of the officials and used to quantify resources tied to the targeted patients.

### Classification of costs

Costs were categorized into capital and recurrent costs. Capital costs were those incurred on items that last longer than one year and recurrent if spent on items that were used up in the year [17]. The recurrent cost items were further classified as personnel (salaries), materials and supplies (medicines, cleaning materials, stationeries, media, etc.), and utilities (electricity, water, gas, phone calls, etc.). Costs for Micro puncture set [Neph set], Amplatz wire, dilators, and catheter nephrostomy drainage were also classified as recurrent because they were single-use items. The finance and accounting department was consulted to get the salary scales of identified personnel and bills for utilities. The costs for materials and supplies came from the procurement department. Capital costs were also further categorized into buildings, equipment and implements, and furniture. Buildings were tape-measured to estimate the space area used for services associated with PCN. The construction costs of the buildings were used to estimate the value of the space used. All equipment and furniture in each cost center were identified and counted and most had receipts indicating the original purchase price. Otherwise, we used replacement prices to estimate their values.

### Description of the cost centers and cost items identified

#### Emergency department

The Emergency Department is attached to the administration block with 3,000 square meters. Identified equipment includes a BP machine, thermometers, and vaginal speculum. Furniture included beds, tables, file cabinets, and chairs. Healthcare workers include a specialist, medical and nurse officers, an assistant nurse officer, enrolled nurses, and a nurse attendant. Materials and supplies included cotton wools, syringes, IV cannulas, methylated spirit, and test tubes. The whole department attends 4,500 patients per month.

#### Central Pathology Laboratory

The central pathology laboratory has 768 square meters and receives about 240,000 samples per month. These samples are coded, hence it was difficult to identify those collected from patients with obstructive uropathy. However, four blood samples were usually drawn from the patient with obstructive uropathy at the emergency department, block-21, and before and after the procedure. The machines used were the chemistry analyzer and the cell-dyne Ruby. The furniture includes tables, file cabins, and chairs.

#### Sewahaji Block-ward 21

The Sewahaji block has 8 wards with a total surface area of 5,231 square meters. Ward 21, which has 360 square meters is where patients with cervical cancer were admitted. The ward is furnished with beds, chairs, tables, and file cabins. An average of 80 patients are admitted to this ward in a month. Equipment identified here included a BP machine, Thermometers, Vaginal speculum, Test tubes, and sample carrier. Healthcare workers include the specialist, medical officer, nurse officer, assistant nurse officer, enrolled nurse, and nurse attendant. The specialist and medical officer spend about 10% of their time in this ward. Materials and supplies included cotton wools, gauzes, syringes of various sizes, normal saline, an IV giving set, IV cannulas, plasters, and methylated spirit.

#### Pharmacy department

The pharmacy was a small office space with 17 square meters also housed within the Sewahaji block. It offers services to about 62,151 patients in a year. Upstream, MNH has a sub-store and main store pharmacies that supplies the block pharmacies, but their costs were not included in the estimation of PCN costs as could only contribute a negligible proportion of the total costs. It was furnished with tables, shelves and chairs, a telephone, and a Refrigerator to keep some medicines in cold temperatures. One pharmacist and a technician worked full-time in the pharmacy.

#### Interventional radiology unit

The Interventional radiology (IR) unit is within the radiology building with 375 square meters. The unit has the following equipment: a fluoroscopy C-arm machine, ultrasound machine, suction machine, and vitals monitor. It is furnished with the following types of furniture: chairs, tables, cabinets, and beds. It also has a telephone. Based on the data from the patient register, an average of 4 cervical cancer patients with obstructive uropathy are treated in a month. Personnel includes a radiologist, radiographer, nurse, and nurse attendant. Materials and supplies included Micro puncture set [Neph set], Amplatz wire 80 cm, 8 F dilators, 10 F dilators, Catheter nephrostomy drainage 10FR,25 cm, Bag drainage 600mls, Iodinated contrast, 3-way stopcock, Nylon suture, Povidone, Lidocaine 2%, Disposable mask, IV Cannula, Disposable cap, surgical gloves, Ultrasound gel, methylated spirit, special X-ray tray, syringes, normal saline, Plasters, Surgical blades, Probe cover, and C-arm cover.

### Data analysis

Cost data were managed and analyzed in Excel spreadsheets (Microsoft Corporation). The costs were collected in local currency (Tanzanian shilling i.e., TZS) and converted to USD using an exchange rate of 1 USD = 2,300 TZS. The useful life of buildings was considered to be 50 years and for other capital items, we assumed it to be 5 years. The equipment’s life years came from the available literature when information could not be obtained from the hospital records. A 3% discount rate was used to calculate the cost of depreciable capital items. The annual costs of capital items were annuitized using the straight-line depreciation method in which the cost of the asset is written off equally during its useful life years.

For each personnel involved, the average time spent on a cervical cancer patient with obstructive uropathy was first determined based on the time spent in the specific cost center by tracking their movement or by interview. For example, the medical specialist spent 10% of his time in ward 21, while the IR specialist spent 100% of his time in the IR unit. Then the ratio of the number of patients requiring PCN to the total number of patients that visited the cost center per month/year was used as the allocation factor. The number of lab samples or prescriptions was used as the basis for allocating the lab and pharmacy personnel costs. The usual working hours were 8 per day over 260 days in a year. Total cost was the sum of capital and recurrent costs that accrued for a year. Unit cost was calculated by dividing total costs by the number of patients treated annually. One-way sensitivity analysis was done by adjusting unit cost for each category of cost items by 10% and 20% upward and downward to identify the most influential cost items. The total cost was based on data collected for the financial year June 2020 to July 2021.

## Results

The total cost of image-guided percutaneous nephrostomy for cervical patients with obstructive uropathy at Muhimbili National Hospital was 18,260.0 USD in a year. This results in a unit cost of 380.4 USD per patient (Table 1). Recurrent costs were estimated to be 17,925,3 USD, which represents 98.2% of the total cost. The IR unit consumed the largest cost 15,239.5 USD, which represents 83.5% of the total cost.

**Table 1:** Cost of performing percutaneous nephrostomy

	Radiology	Pharmacy	Sewahaji	Emergency	CPL Lab	Total
*Recurrent costs*						
Personnel	613.8	0.9	1,173.1	42.2	0.9	1,830.9
Materials & supplies	14,366.7	541.4	47.6	468.5	0.0	15,424.2
Utilities	40.2	0.2	554.8	73.9	1.1	670.2
Sub-total	15,020.7	542.5	1,175.5	584.6	2.0	17,925.3
*Capital costs*						
Building	26.2	0.1	83.7	21.3	1.1	132.4
Equipment	190.2	0	0.7	0	0.1	191.0
Furniture	2.4	0.1	8.4	0.1	0.0	11.0
Sub-total	218.8	0.2	92.8	21.4	1.2	334.4
Total cost	15,239.5	542.7	1,868.3	606.0	3.2	18,259.7

**Key**: CPL-Central Pathology Laboratory

**Fig. 2:**
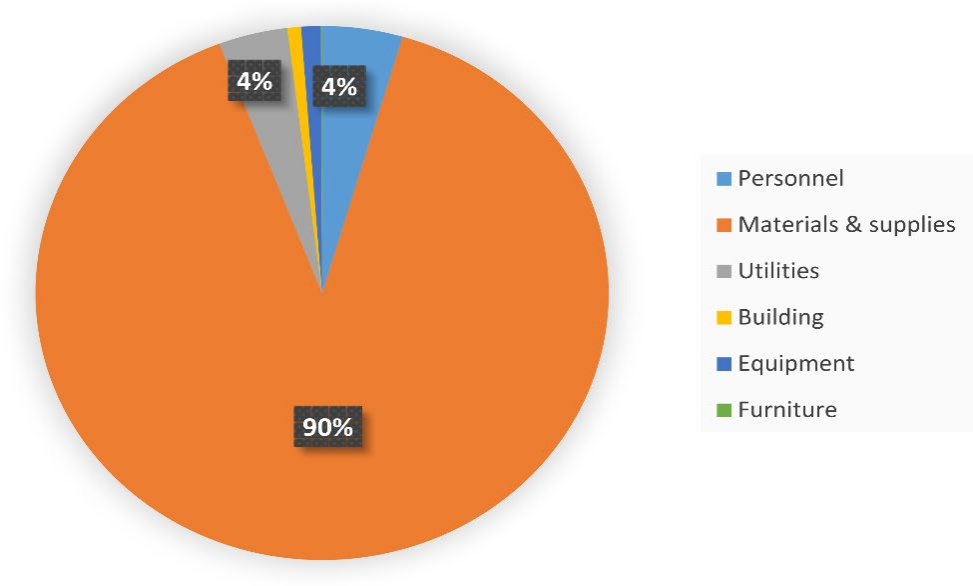
Cost drivers

As indicated in Fig. 2, the cost of materials and supplies particularly from the Interventional Radiology department accounted for the largest share of the total costs. The most expensive items here were the Catheters Nephrostomy Drainage 10FR, 25 cm at 119.2 USD, Amplatz guide wire 80 cm at 75.2 USD, and Micro-puncture set at 27.7 USD. Other expensive items were the 8 and 10 F dilators each at about 20 USD, and the Probe cover and C-arm cover at 8 and 10.8 USD, respectively. These are single-use items.

**Fig. 3:**
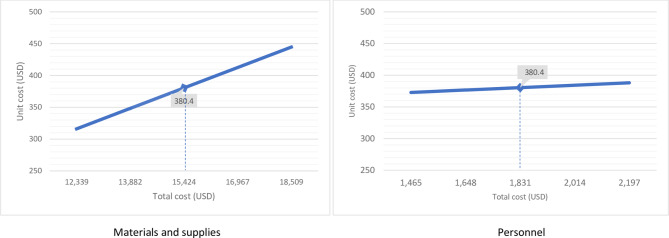
Changes in unit cost versus changes in input costs. The dotted lines represent the mean cost, while the data points left and right of the dotted line represent ± 10% and ± 20% around the mean

We further conducted one-way sensitivity analyses to determine how the estimated unit cost responds to changes in input costs. Unit cost was most sensitive to materials and supplies costs. Figure 3 shows that an increase in materials and supplies costs by 10% and 20% increased the unit cost to 412.5 USD and 444 USD, respectively. A similar percentage decrease in materials and supplies reduced the unit costs to 348.3 USD and 316.1 USD, respectively. Also, an increase in personnel cost by 10% and 20% increased the unit cost slightly to 384.2 USD and 388.0 USD, respectively. A similar percentage decrease in personnel cost reduced the unit cost to 376.6 USD and 372.8 USD, respectively.

**Fig. 4:**
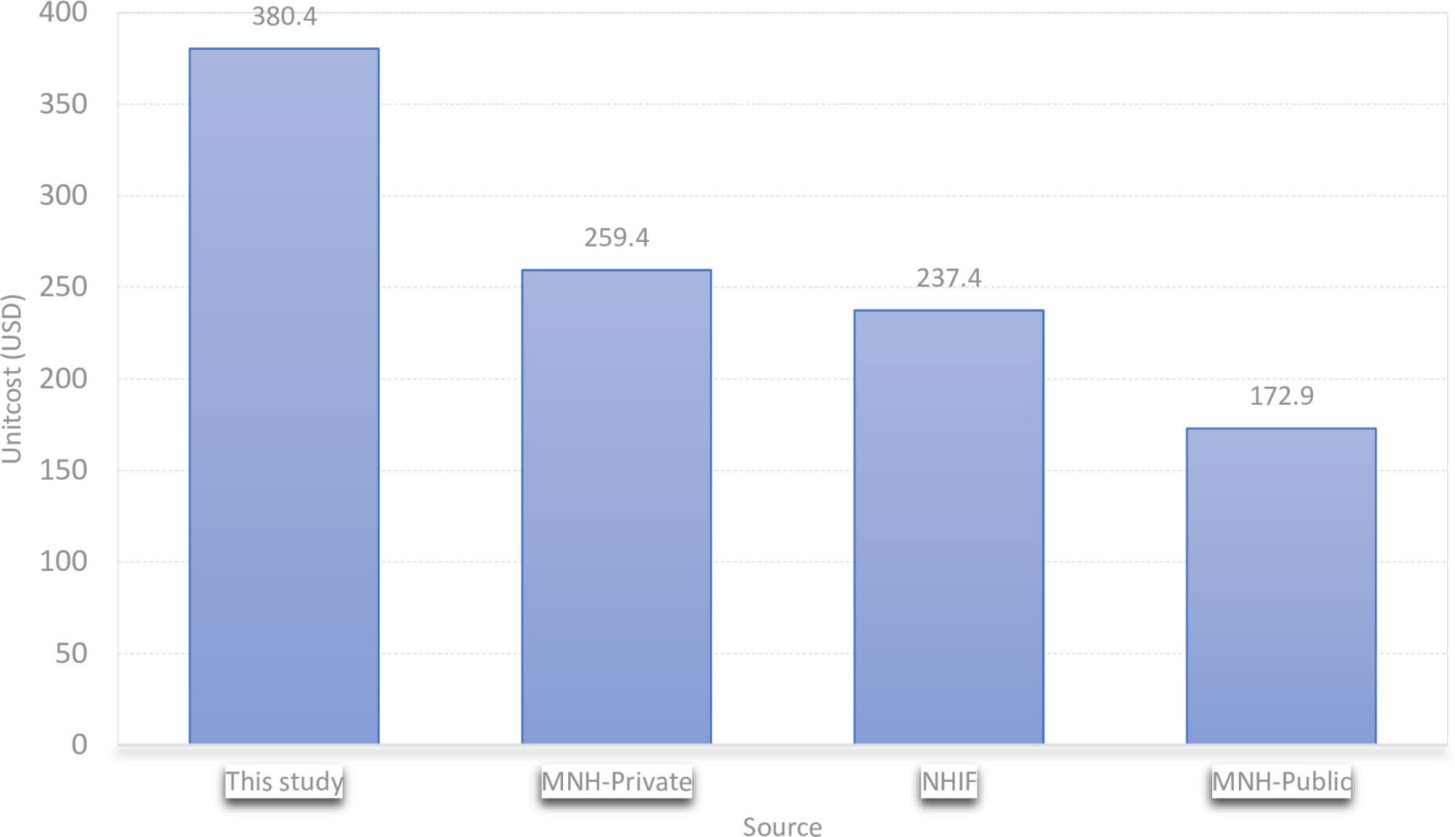
Comparison between the estimated cost, and NHIF and MNH reference prices

Figure 4 compares the estimated unit cost of PCN, the NHIF reimbursement price and the fees currently charged by Muhimbili National Hospital for private and public patients. The cost of 380.4 USD which was estimated by this study was higher than the NHIF reimbursement cost of 237.4 USD per patient and the fee of 259.4 USD and 172.9 USD per patient for private and public patients, respectively, charged by Muhimbili National Hospital.

## Discussion

This study found that the unit cost of providing the newly introduced percutaneous nephrostomy to cervical cancer patients with obstructive uropathy at Muhimbili National Hospital was USD 380.4 USD. This is an expensive procedure in a country where per capita health expenditure is USD 40.5 and the out-of-pocket health expenditure accounts for 32% of total health expenditure [18]. The estimated unit cost is three times higher than the minimum monthly government wage and more than four times that in the private sector [19].

Few studies have estimated the cost of managing cervical cancer in Tanzania. The study by Chuwa et al. (2014) showed that the overall cost of treating cervical cancer at Ocean Road Cancer Institute was USD 2,452.5 per patient [20]. The mean direct outpatient cost was 1,039.5 USD (66.7% due to radiotherapy cost) and the inpatient direct cost was 2,301.8 USD (52.4% due to admission cost) [20]. The study by Nelson et al. (2016), in the same hospital, also found that the average screening and treatment costs were 2,526 USD and 2,482 USD for patients receiving cancer screening and for unscreened patients, respectively [21]. In the study by Santos et al. in Brazil, the cost of invasive cervical cancer treatment was USD 2,219.7 per patient, of which the direct medical costs accounted for 81.2% of the total value, of which radiotherapy and outpatient chemotherapy had the largest share [22].

As for direct non-medical costs and indirect costs for outpatient cervical cancer care in Tanzania, the cost has been estimated at 827.5 USD (55% due to food) and 374.3 USD for inpatient care, of which 47% was accounted for food expenditure followed by 43% due to lost working days [20]. In Ethiopia, cervical cancer was also found to have large patient costs i.e., the average outpatient cost was estimated at 407.2 USD per patient, of which direct medical costs accounted for 82% and indirect costs 18%, while inpatient cost was 404.4 USD per patient, of which direct medical cost was 74% and 26% were non-medical costs [23]. Another study from Brazil also showed that indirect costs due to cervical cancer represented 51% of the total patient costs, followed by direct medical costs (visits and procedures) at 41% and direct non-medical costs (transportation) at 8% [24].

Recently, the WHO released a report showing the cost estimates to support the national response to cervical cancer. The report showed that the economic cost of a fully immunized girl with HPV was 16.8 USD, screening 4.5 USD, and 11.5 USD for precancer treatment [25]. The costs of preventing cervical cancer are far much less than the costs of managing the disease and its associated complications as this study shows. Therefore, vaccination against HPV and screening should be expanded to reduce morbidity and mortality and subsequent treatment costs that are high for patients without health insurance.

The economic data presented here also show that materials and supplies specific to PCN and to some extent personnel costs were the cost drivers. The most expensive materials and supplies included single-use items such as Catheters Nephrostomy Drainage 10FR, 25 cm, Amplatz guide wire 80 cm, Micro-puncture set, 8 F dilator and 10 F dilator, the Probe, and C-arm covers. The study conducted in Vietnam on medical costs for the treatment of cervical cancer found that medical equipment had the largest share of the total cost for treatment unlike this study [26]. The differences may be due to the organization and structure of the healthcare system and the type of equipment in use. The proportion of advanced cervical cancer patients with obstructive uropathy at Muhimbili National Hospital is quite small, which could be the reason for the relatively small share of equipment use.

The evidence provided by this study is useful to inform pricing decisions and scale-up of the intervention. Cost information could also be used to populate cost-effectiveness models comparing PCN with the standard of care. Currently, image-guided PCN in Tanzania is only available at Muhimbili National Hospital. Therefore, it is important to build the capacity of other hospitals at regional and district levels to increase access and reduce direct non-medical patient costs including transportation and loss in productivity. Apart from the identified resources, short training in human resources will be required, which could be done by experts already at Muhimbili National Hospital.

The estimated unit cost was higher than the reimbursement cost offered by the National Health Insurance Fund (NHIF), and the fees charged by Muhimbili National Hospital (MNH) for private and public patients. The implication of MNH charging less than the actual resource use is that MNH is operating at a loss, hence draining its scarce health resources. Therefore, this underscores the importance of conducting costing and cost-effectiveness studies to inform pricing and reimbursement decisions.

### Strengths and limitations

Firstly, to the best of our knowledge, this is the first study to estimate the cost of percutaneous nephrostomy in Tanzania, which can help to inform resource allocation decisions. Secondly, the study used a micro-costing approach which offers the advantages of accuracy and transparency on resource use compared to other methods. Nevertheless, our results should be interpreted in the context of several limitations. Firstly, the study did not include costs of training, supervision, administration, and fringe benefits payable to health workers including vacations and meetings, sick leave, and overtime. These were omitted because data were not readily available or were difficult to measure. Secondly, Muhimbili is a National Hospital; hence it is not only large but also has a complex organizational structure rendering it difficult to track every resource item with the required precision. Some simplifying assumptions were applied such as omitting overhead costs, which might have underestimated the actual amount of resources consumed and the resulting unit cost.

## Conclusions

Image-guided PCN is a relatively expensive procedure for a typical patient without health insurance. The cost was almost ten-fold higher than the per capita health expenditure, three times and four times higher than the minimum monthly government and private sector wages, respectively, in Tanzania. The estimated cost was higher than the cost charged to the private and public patients and the NHIF reimbursement cost, which underscores the importance of conducting costing studies to inform pricing and reimbursement decisions.

## Data Availability

The datasets used and analyzed during the current study are available from the corresponding author upon reasonable request.

## List of Abbreviations

CPL: Central Pathology Laboratory
HMIS: Health Management and Information System
HPV: Human Papilloma Virus
IR: Interventional Radiology
MNH: Muhimbili National Hospital
NCCP: National Cancer Control Program
NHIF: National Health Insurance Fund.
ORCI: Ocean Road Cancer Institute
PCN: Percutaneous Nephrostomy

## References

[1] WHO (2014). Comprehensive Cervical Cancer Control: a guide to essential practice.

[2] Bruni L, Albero G, Serrano B, Mena M, Collado JJ, Gómez D et al. Human papillomavirus and related Diseases in the World. Summary Report 22 October 2021. Barcelona: ICO/IARC Information Centre on HPV and Cancer (HPV Information Centre).

[3] Bray F, Ferlay J, Soerjomataram I, Siegel RL, Torre LA, Jemal A (2018). Global cancer statistics 2018: GLOBOCAN estimates of incidence and mortality worldwide for 36 cancers in 185 countries. CA Cancer J Clin.

[4] Ferlay J, Ervik M, Lam F, Colombet M, Mery L, Piñeros M (2020). Global Cancer Observatory: Cancer Today.

[5] WHO (2020). Costing the National Response to Cervical Cancer: United Republic of Tanzania, 2020–2024.

[6] Souza AC, Souza AN, Kirsztajn R, Kirsztajn GM (2016). Cervical cancer: renal complications and survival after percutaneous nephrostomy. Rev Assoc Med Bras (1992).

[7] Mishra K, Desai A, Patel S, Mankad M, Dave K (2009). Role of percutaneous nephrostomy in advanced cervical carcinoma with obstructive uropathy: a case series. Indian J Palliat Care.

[8] Patel K, Foster NR, Kumar A, Grudem M, Longenbach S, Bakkum-Gamez J (2015). Hydronephrosis in patients with cervical cancer: an assessment of morbidity and survival. Support Care Cancer.

[9] Sood G, Sood A, Jindal A, Verma DK, Dhiman DS (2006). Ultrasound guided percutaneous nephrostomy for obstructive uropathy in benign and malignant diseases. Int Braz J Urol.

[10] Mlange R, Matovelo D, Rambau P, Kidenya B (2016). Patient and disease characteristics associated with late tumour stage at presentation of cervical cancer in northwestern Tanzania. BMC Womens Health.

[11] Bruni L, Albero G, Serrano B, Mena M, Gómez D, Muñoz J et al. ICO/IARC information centre on HPV and cancer (HPV information centre). Human papillomavirus and related diseases in the world. Summary Rep 2019 Jun 17. 2019;17(6).

[12] Pergialiotis V, Bellos I, Thomakos N, Haidopoulos D, Perrea DN, Kontzoglou K (2019). Survival outcomes of patients with cervical cancer and accompanying hydronephrosis: a systematic review of the literature. Oncol Rev.

[13] Ali SM, Mehmood K, Faiq SM, Ali B, Naqvi SA, Rizvi AU (2013). Frequency of complications in image guided percutaneous nephrostomy. J Pak Med Assoc.

[14] World Population Review. https://worldpopulationreview.com/world-cities/dar-es-salaam-population. 2021.

[15] Gwaleba M, Masum F (2018). Participation of Informal settlers in Participatory Land Use Planning Project in Pursuit of Tenure Security. Urban Forum.

[16] Sach TH, Desborough J, Houghton J, Holland R (2015). Applying micro-costing methods to estimate the costs of pharmacy interventions: an illustration using multi-professional clinical medication reviews in care homes for older people. Int J Pharm Pract.

[17] Shepard D, Hodgkin D, Anthony Y (2000). Analysis of hospital costs: a manual for managers.

[18] MoH (2022). National Health Accounts for the years 2017–2020.

[19] National Bureau of Statistics. Tanzania in Fig. 2021. Dodoma: Ministry of Finance and Planning; 2022. p. https://www.nbs.go.tz/index.php/en/tanzania-in-figures/784-tanzania-in-figures-2021.

[20] Chuwa H, Sakafu L, Ngoma T (2020). Descriptive prospective cohort study at Ocean Road Cancer Institute, Tanzania to Estimate the total cost of Cervical Cancer Management. Adv Res J Cancer.

[21] Nelson S, Kim J, Wilson FA, Soliman AS, Ngoma T, Kahesa C (2016). Cost-effectiveness of screening and treatment for Cervical Cancer in Tanzania: implications for other Sub-Saharan African Countries. Value Health Reg Issues.

[22] Santos CL, Souza AI, Figueiroa JN, Vidal SA (2019). Estimation of the costs of Invasive Cervical Cancer Treatment in Brazil: a Micro-Costing Study. Revista brasileira de ginecologia e obstetricia: revista da Federacao Brasileira das Sociedades de Ginecologia e Obstetricia.

[23] Hailu A, Mariam DH (2013). Patient side cost and its predictors for cervical cancer in Ethiopia: a cross sectional hospital based study. BMC Cancer.

[24] Novaes HM, Itria A, Silva GA, Sartori AM, Rama CH, Soárez PC (2015). Annual national direct and indirect cost estimates of the prevention and treatment of cervical cancer in Brazil. Clin (Sao Paulo).

[25] WHO (2020). Costing the National Response to Cervical Cancer: United Republic of Tanzania 2020–2024.

[26] Nguyen AD, Hoang MV, Nguyen CC (2018). Medical costs for the treatment of cervical cancer at central hospitals in Vietnam. Health Care Women Int.

